# Association between fish and shellfish consumption, n-3 polyunsaturated fatty acids, and gastric cancer risk: the Japan Public Health Center-based Prospective Study

**DOI:** 10.1007/s00394-024-03343-9

**Published:** 2024-05-04

**Authors:** Mayo Hirabayashi, Calistus Wilunda, Utako Murai, Taiki Yamaji, Motoki Iwasaki, Manami Inoue, Shoichiro Tsugane, Norie Sawada, for the JPHC Study Group

**Affiliations:** 1 Division of Prevention, Institute for Cancer Control, National Cancer Center, 5-1-1 Tsukiji, Chuo-ku, Tokyo, 104-0045 Japan; 2 Division of Cohort Research, Institute for Cancer Control, National Cancer Center, Tokyo, Japan; 3 Division of Epidemiology, Institute for Cancer Control, National Cancer Center, Tokyo, Japan; 4 African Population and Health Research Center, Nairobi, Kenya; 5 Graduate School of Public Health, International University of Health and Welfare, Tokyo, Japan

**Keywords:** Gastric cancer, *Helicobacter pylori*, Fish consumption, n-3 PUFAs, The JPHC Study

## Abstract

**Purpose:**

Fish and shellfish consumption is suggested to be a cancer-protective factor. However, studies investigating this association for gastric cancer, especially considering *Helicobacter pylori* (*H. pylori*) and atrophic gastritis (AG), are limited. We investigated gastric cancer risk associated with fish, shellfish, and n-3 polyunsaturated fatty acids (n-3 PUFAs) consumption among Japanese adults.

**Methods:**

90,504 subjects enrolled in the Japan Public Health Center-based Prospective Study (JPHC Study) were followed until December 2013. Dietary intake data were collected using a food frequency questionnaire. Hazard ratios (HRs) and 95% confidence intervals (CIs) were calculated for gastric cancer risk associated with fish and shellfish consumption and marine n-3 PUFAs (sum of eicosapentaenoic acid (EPA), docosapentaenoic acid (DPA), and docosahexaenoic acid (DHA)) using Cox proportional hazards models. Among those with avaliable data, we conducted a subgroup analysis taking *H. pylori* infection and AG status  into consideration.

**Results:**

There were 2,701 gastric cancer cases during an average of 15 years of follow-up. We observed an increased gastric cancer risk for salted fish consumption for men [HR for fifth quintile versus first quintile 1.43 (95% CI 1.18–1.75)] and for women [HR 1.33 (95% CI 1.00–1.77)]. We observed a weak risk reduction trend for women as the intake of marine n-3 PUFAs increased (*p-*trend:0.07). When we included *H. pylori* infection and atrophic gastritis status in the analysis, the associations diminished.

**Conclusion:**

Our results suggest that salted fish increases gastric cancer risk for men and women, while marine n-3 PUFAs marginally decreases this risk among women in Japan.

## Introduction

Gastric cancer is the fifth-most common and fourth-most lethal cancer worldwide, with over 1 million new cases and 770,000 deaths in 2020 [1]. The highest age-standardised incident rates (ASIRs) for gastric cancer, calculated per 100,000 person-years are observed in eastern Asia (32.5 for men; 13.2 for women), where 60% of all gastric cancers occur [1, 2]. Given this high burden, it is crucial to identify modifiable risk and protective factors for gastric cancer for better targeted and more effective prevention.

While *Helicobacter pylori* (*H. pylori*) infection remains the leading cause of gastric cancer, a 2018 report by the World Cancer Research Fund (WCRF) and the American Institute for Cancer Research (AICR) on the relationship between diet and cancer suggested that consumption of certain food, such as foods preserved by salting, processed meat, or consumption of alcoholic drinks may be associated with gastric cancer development [3].

Diet has received attention in the past decades for its potential role in preventing cancer. Seafood is a part of a diet for many: fish, in particular, is a known source of n-3 polyunsaturated fatty acids (PUFAs) and other nutrients such as vitamin D and selenium [3].

Epidemiological studies have found conflicting results on the association between fish consumption and gastric cancer [4–6]. Dietary n-3 PUFAs are reported to suppress mutations, inhibit cell growth, enhance cell apoptosis, and ultimately reduce cancer risk [7]. On the other hand, salted fish, fish that is treated with a combination of brining, dry salting, or pickle curing, have been reported to have a dose-dependent relation with gastric cancer risk [8]. Moreover, grilled or charbroiled fish can contain mutagenic and carcinogenic heterocyclic amines and polycyclic aromatic hydrocarbons, which may increase gastric cancer risk [9, 10]. While the WCRF/AICR reported that high-salt foods and salt-preserved foods have a probable association with gastric cancer risk, the same report concluded that there was limited evidence on the association between gastric cancer and consumption of processed, grilled, or charbroiled fish [3]. A 2011 meta-analysis with 15 case–control studies and two cohort studies [11] found no association between fish consumption and gastric cancer risk (relative risk (RR) 0.87, 95% confidence interval (CI) 0.71–1.07). A 2014 meta-analysis [12] with 27 prospective cohort studies found an inverse association between fish consumption and gastrointestinal cancer risk; however, when they analysed seven cohorts with available data on gastric cancer, 20 grams (g) per day increment of fish slightly increased gastric cancer risk (RR 1.03, 95% CI 1.00–1.05).

The gastric cancer risk associated with fish consumption, other than salted fish roe and fish preserved by salt, remains unclear. Furthermore, limited studies have taken *H. pylori* infection into consideration while assessing the association between fish and shellfish consumption, n-3 PUFAs, and gastric cancer risk.

Therefore, the aim of this study was to investigate the association between fish and shellfish consumption, and n-3 PUFAs and gastric cancer risk using a Japanese population-based prospective study.

## Methods

### Study design

The Japan Public Health Center-based Prospective Study (the JPHC Study) is an ongoing cohort study designed to investigate associations between lifestyle habits and non-communicable diseases. The JPHC Study has been described in detail elsewhere [13]. Briefly, the JPHC Study consists of Cohort I, established in 1990, and Cohort II, established in 1993, involving a total of 140,420 individuals (68,722 men, 71,698 women) in 11 public health centres (PHC) nationwide aged 40–69 at the beginning of the baseline survey.

### Study population

**Fig. 1:**
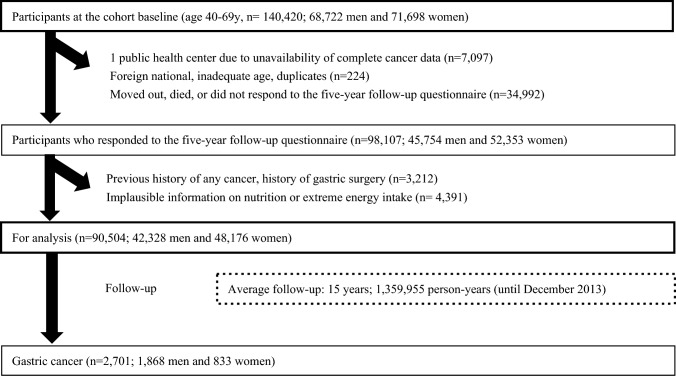
Participant flowchart

For this study, we included those who responded to a self-administered 5-year follow-up questionnaire between 1995 and 1999, which included comprehensive information on dietary intake and lifestyle-related factors. Figure 1 shows the flow diagram of study participant selection. We excluded participants from Katsushika, Tokyo, due to the lack of cancer incidence information. After excluding those who died or moved out of the study area before the 5-year follow-up survey, non-Japanese nationals, incorrect birthdate, duplicates, cancer diagnosis before the 5-year follow-up survey, non-response, history of any type of cancer, history of gastric surgery, or reported extreme energy intake (men: < 800 or > 4200 kcal; women: < 500 or > 3500 kcal), 90,504 (42,328 men and 48,176 women) participants remained for analysis.

### Exposure and covariates

The self-administered questionnaire at the 5-year follow-up collected data on smoking, alcohol consumption, physical activity, anthropometry, and medical history. It also included a comprehensive food frequency questionnaire (FFQ). It covered 138 food and drink items with nine eating frequency categories (never; 1–3 times/month; 1–2 times/week; 3–4 times/week; 5–6 times/week; once/day; 2–3 times/day; 4–6 times/day; ≥ 7 times/day) and three portion sizes (small: 50% smaller than the standard size; medium: standard; large: 50% larger than the standard size). The FFQ also included questions on consumption of 19 seafood items (from here on referred to as fish and shellfish: canned tuna, salmon/trout, bonito/tuna, cod/flatfish, sea bream, horse mackerel/sardines, mackerel pike/mackerel, *shirasuboshi* (dried young sardines), *chikuwa* (Japanese fish cake), *kamaboko* (Japanese cured surimi (minced fish paste)), salted fish, salted fish roe, dried fish, eel, squid, octopus, prawn, short-necked clam, and viviparide). These items were further categorised into: fish (canned tuna, salmon/trout, bonito/tuna, cod/flatfish, sea bream, horse mackerel/sardines, mackerel pike/mackerel, *shirasuboshi*, salted fish, dried fish, and eel), salted fish (salted pike/mackerel, salted cod/flatfish, salted salmon/trout, salted fish roe, dried fish, and *shirasuboshi*), and n-3 PUFAs-rich fish (salmon/trout, horse mackerel/sardines, mackerel pike/mackerel, eel, and sea bream, based on the mount of n-3 PUFAs in 100 g (g) edible fish portion). Daily consumption of fish and shellfish, salted fish, and n-3 PUFA-rich fish (measured in g/day) were calculated by multiplying the frequency by relative portion size. For n-3 PUFAs, we focused on eicosapentaenoic acid (EPA), docosapentaenoic acid (DPA), and docosahexaenoic acid (DHA), n-3 PUFAs that are often found high in marine food, to look at the association between marine-based n-3 PUFAs and gastric cancer risk [14]. Daily intake of EPA, DPA, and DHA was calculated using Japanese food fatty acid composition tables [15]. The sum of EPA, DPA, and DHA was considered as marine n-3 PUFAs in our study, as about 90% of these n-3 PUFAs consumed among the study participants derived from marine food. All dietary intake variables were log-transformed and adjusted for total energy intake using the residual method and divided into quintiles for both sexes.

FFQ validation for fish,shellfish, and marine n-3 PUFAs were conducted using 14- or 28-day dietary records, which are considered a gold standard. Spearman correlation coefficient for fish and shellfish for Cohort I was men 0.32, women 0.32, and for Cohort II, men 0.27, women 0.23 [16, 17]. Spearman correlation coefficient for marine n-3 PUFAs in Cohort I were EPA: men 0.38, women 0.45; DPA: men 0.32, women 0.39; and DHA: men 0.34, women 0.37 [18]. Reproducibility of the FFQ was evaluated by administering two questionnaires 1 year apart. Spearman correlation coefficient for fish and shellfish for Cohort I was men 0.44, women 0.34, and for Cohort II, men 0.45, women 0.40 [17, 19].

### Laboratory analysis

Information on *H. pylori* infection and atrophic gastritis was available for 31% of participants who provided blood samples at the baseline survey in Cohort II (8,702 men and 15,672 women). *H. pylori* was measured using plasma immunoglobin G (IgG) level through an enzyme immunoassay (Eiken Kagaku, Tokyo, Japan). IgG titer ≥ 10 U/mL was considered as *H. pylori-*positive. Atrophic gastritis was defined using plasma levels of pepsinogen I and II, measured by a latex-agglutination assay (Eiken Kagaku, Tokyo, Japan), with pepsinogen I ≤ 70 ng/mL and pepsinogen I/II ratio ≤ 3.0 considered as positive [20].

### Follow-up and identification of gastric cancer case

Person-years of follow-up were calculated from the date of the 5-year follow-up survey to the date of gastric cancer diagnosis, death, move out from the study area, or until 31 December 2013 (except Osaka PHC: 31 December 2012), whichever came first. The residential registry was used to confirm residence and survival status. Gastric cancer incidence was identified by active patient notification from major local hospitals in each PHC area and linkage of the record with population-based cancer registries, and were supplemented by death certificates. Gastric cancer was coded using the International Classification of Diseases (ICD) for Oncology, 3rd edition (C16.0 to 16.9) [21]. Residual cases were tumours that could not be classified due to overlapping lesions (C16.8) or no information (C16.9).

### Statistical analysis

We used Cox proportional hazards regression models to estimate hazard ratios (HRs) and their 95% confidence intervals (CIs), running multivariable models using the lowest quintile as a reference. We conducted the analysis separately for men and women since gastric cancer incidence is significantly higher in men [22]. The models were adjusted for potential confounders based on previous studies: metabolic equivalent of tasks (METs, continuous), body mass index (BMI, weight (kg)/height(m)^2^, continuous), total energy, meat consumption (energy-adjusted, continuous), fruit consumption (energy-adjusted, continuous), vegetable consumption (energy-adjusted, continuous), alcohol consumption (main analysis: men: never/former, < 150, 150–299, 300–449, ≥ 450 g ethanol/week; women: never/former, < 150, 150–299, ≥ 300 g ethanol/week; *H. pylori* and atrophic gastritis analysis: men: never/former, < 150, 150–299, 300–449, ≥ 450 g ethanol/week; women: never/former, current), smoking (never, former, < 20 cigarettes/day, ≥ 20 cigarettes/day), history of diabetes mellitus, use of anti-cholesterol drugs, history of gastric ulcer, and family history of gastric cancer. We used two definitions of salted food depending on the analysis: salted food *excluding* salted fish, or salted food *and* salted fish. When evaluating the association between salted fish and gastric cancer, we used salted food *excluding* salted fish (energy adjusted, continuous). For the analysis on the association between fish other than salted fish and gastric cancer, we used salted food *and* salted fish (energy adjusted, continuous) to evaluate the effect of salt. Because the consumption of salted food and salted fish varies heavily by the region of Japan [23], we thought that adjusting the model by PHC may mask the true association between fish consumption and gastric cancer. Therefore, we included PHC as a covariate for all models except for salted fish. We tested for linear trends using the median value of each quintile. For salted fish, fish excluding salted fish, and n-3 PUFAs-rich fish analyses, we adjusted for fish excluding salted fish, salted fish, and lean fish consumption, respectively, in addition to the confounding variables listed above. We performed sensitivity analysis by running the same model excluding gastric cancer cases diagnosed within 3 years of follow-up. For multi-variable analysis, we used multiple imputation to impute missing data on METs (3.6%), alcohol consumption (7.7%), smoking status (4.9%), BMI (2.6%), family history of gastric cancer (12.5%), and vegetable intake (0.1%) using the command “mi estimate”, created based on Rubin rules [24]. We assessed interaction between the exposure variables and BMI (< 25, ≥ 25 kg/m^2^), smoking (never/former, current), and alcohol consumption (never/former, ever) by comparing the model with or without an interaction term using the likelihood ratio test with or without an interaction term. We also conducted an analysis based on the anatomical region of the tumour (proximal, upper one-third of the stomach, versus distal, lower two-thirds of the stomach), based on The Japanese classification of gastric carcinoma, 3rd edition [25].

Among those with available information on *H. pylori* and atrophic gastritis, we performed subgroup analyses on the association between fish and shellfish consumption and risk of gastric cancer based on the *H. pylori* infection and atrophic gastritis status. Given that atrophic gastritis is caused by *H. pylori* infection-related chronic gastritis [26, 27] and the small number of cases, we divided the subjects into four categories based on their *H. pylori* infection and atrophic gastritis status: *H. pylori* antibody and atrophic gastritis negative (*H. pylori − */AG −), *H. pylori* antibody positive (*H. pylori* +), those with atrophic gastritis (AG +), and *H. pylori* antibody and/or atrophic gastritis positive (*H. pylori* + and/or AG +).

All analyses were performed using Stata 16.0 (StataCorp LLC).

## Results

During an average of 15 years of follow-up, 2701 gastric cancer cases were diagnosed (1868 men and 833 women). Table 1 shows the baseline characteristics of study subjects by fish and shellfish consumption quintile. Compared to the lowest quintile, the higher quintile subjects were older, more likely to have a history of diabetes mellitus, and consumed less alcohol.

**Table 1:** Basic characteristics of study participants

	Men (*n* = 42,338)			Women (*n* = 48,176)		
	Q1 (*n* = 8466)	Q3 (*n* = 8465)	Q5 (*n* = 8,465)	Q1 (96236)	Q3 (9635)	Q5 (9635)
Fish and shellfish^*^ (g/day), median	26.9	63.7	128.6	34.0	78.6	152.6
Gastric cancer, *n*	347	379	406	145	180	165
Proximal gastric cancer, *n*	39	47	55	12	11	13
Distal gastric cancer, *n*	222	249	251	92	123	118
Age (years), mean ± SD	57.0 ± 7.9	57.7 ± 7.8	59.7 ± 7.8	58.5 ± 8.4	57.7 ± 7.8	59.6 ± 7.7
BMI (kg/m^2^), mean ± SD	23.7 ± 2.9	23.5 ± 2.8	23.5 ± 2.9	23.7 ± 3.3	23.4 ± 3.1	23.6 ± 3.2
Current smoker, %	48.8	49.0	45.3	6.3	5.8	5.6
Alcohol consumption						
Never/former drinker, %	25.5	27.2	37.4	84.0	81.3	84.5
Mean ethanol consumption (g/week)	273.5	193.1	135.9	17.8	13.6	9.8
History of diabetes mellitus, %	7.1	8.2	12.0	3.8	4.1	5.4
History of gastric ulcer, %	4.6	5.0	4.8	1.7	2.0	2.1
Family history of gastric cancer, %	5.8	6.8	6.8	4.5	7.5	6.7
Dietary intake (IQR)						
Energy, kcal/day	2240 (1762–2763)	2106 (1726–2558)	1832 (1482–2253)	1767 (1374–2232)	1764 (1431–2202)	1684 (1362–2057)
Salted food, g/day	29.1 (15.9–48.50)	35.9 (22.1–55.1)	40.3 (24.8–61.2)	30.4(16.5–52.8)	41.5(24.2–68.8)	46.9(28.9–73.6)
n-3 PUFAs-rich fish, g/day	8.31 (4.65–12.3)	22.8 (16.9–29.0)	52.2 (37.1–74.0)	10.1 (5.4–14.7)	28.2 (22.5–34.8)	65.6 (51.2–89.7)
Marine n-3 PUFAs, g/day	0.36 (0.25–0.46)	0.82 (0.69–0.96)	1.65 (1.32–2.11)	0.39 (0.28–0.48)	0.94 (0.88–1.02)	1.92 (1.69–2.38)
EPA, g/day	0.11 (0.07–0.15)	0.27 (0.23–0.33)	0.56 (0.44–0.73)	0.12 (0.84–0.16)	0.32 (0.30–0.35)	0.68 (0.60–0.84)
DPA, g/day	0.04 (0.03–0.05)	0.08 (0.71–0.10)	0.16 (0.12–0.20)	0.04 (0.03–0.05)	0.09 (0.08–0.10)	0.18 (0.15–0.21)
DHA, g/day	0.22 (0.16–0.28)	0.49 (0.41–0.56)	0.95 (0.77–1.20)	0.24 (0.18–0.29)	0.55 (0.51–0.59)	1.09 (0.96–1.33)

**Fish and shellfish**: canned tuna, salmon/trout, bonito/tuna, cod/flatfish, sea bream, horse mackerel/sardines, mackerel pike/mackerel, chikuwa (Japanese fish cake), kamaboko (Japanese cured surimi (minced fish paste)), salted fish (salted pike/mackerel, salted cod/flatfish, salted salmon/trout, salted fish roe, dried fish, and shirasuboshi (dried young sardines)), eel, squid, octopus, prawn, short-necked clam, and viviparide

**n-3 PUFAs-rich fish**: salmon/trout, horse mackerel/sardines, mackerel pike/mackerel, eel, and sea bream

**Marine n-3 PUFAs** : Sum of EPA, DPA, and DHA

*Q* quantile, *n* number of cases, *BMI* body mass index, *g* grams, *IQR* interquartile range,* EPA* eicosapentaenoic acid,* DPA* docosapentaenoic acid,* DHA* docosahexaenoic acid

Tables 2 and 3 show gastric cancer risk by consumption of fish and shellfish, fish, fish excluding salted fish, salted fish, n-3 PUFAs-rich fish, and marine n-3 PUFAs for men and women, respectively. There was an increase in gastric cancer risk associated with salted fish consumption for men (HR for fifth quintile versus first quintile 1.43 (95% CI 1.18–1.75); *p-*trend 0.006) (Table 2). Increased gastric cancer risk was also observed in women who consumed salted fish in high quantities (HR for fifth quintile versus first quintile 1.33 (95% CI 1.00–1.77); *p*-trend 0.21) (Table 3). We observed a weak decrease in gastric cancer risk trend for marine n-3 PUFAs (HR for fifth quintile versus first quintile 0.79 (95% CI 0.62–1.04); *p-*trend 0.07) (Table 3). Consumption of fish and shellfish, fish excluding salted fish, or n-3 PUFAs-rich fish was not associated with gastric cancer risk. These findings did not change when we conducted a sensitivity analysis which excluded gastric cancer cases occurring within 3 years of follow-up. We found no interaction between fish and shellfish consumption and any of the exposure variables for both men and women (data not shown).

**Table 2:** Hazard ratios (HR) and 95% confidence intervals (CIs) of gastric cancer risk by fish and shellfish consumption for men

Quintile (Q) of intake	Person-years	Cases (*n*)	Model 1 HR (95% CI)	*p*-trend	Model 2 HR (95% CI)	*p*-trend	Sensitivity analysis HR (95%CI)	*p*-trend
Fish and shellfish								
Q1	124,489	347	1.0 (Ref)	0.77	1.0 (Ref)	0.89	1.0 (Ref)	0.79
Q2	124,551	342	0.91 (0.78–1.05)		0.92 (0.77–1.11)		0.93 (0.77–1.13)	
Q3	124,389	379	0.94 (0.81–1.09)		0.88 (0.73–1.05)		0.90 (0.74–1.10)	
Q4	122,973	394	0.96 (0.83–1.11)		1.03 (0.86–1.23)		1.07 (0.88–1.29)	
Q5	119,674	406	0.94 (0.81–1.09)		0.95 (0.79–1.15)		0.96 (0.79–1.18)	
Per 50 g/day increase			1.00 (0.95–1.05)		1.00 (0.93–1.07)		1.01 (0.94–1.08)	
Fish								
Q1	124,376	352	1.0 (Ref)	0.47	1.0 (Ref)	0.80	1.0 (Ref)	0.75
Q2	125,063	333	0.88 (0.76–1.02)		0.86 (0.72–1.03)		0.84 (0.69–1.02)	
Q3	124,425	386	0.94 (0.82–1.09)		0.89 (0.75–1.07)		0.86 (0.71–1.04)	
Q4	122,588	407	0.98 (0.85–1.13)		0.98 (0.82–1.17)		0.99 (0.83–1.20)	
Q5	119,624	390	0.90 (0.78–1.04)		0.91 (0.75–1.09)		0.88 (0.72–1.08)	
Per 50 g/day increase			0.99 (0.94–1.04)		0.98 (0.91–1.05)		0.98 (0.91–1.06)	
Fish excluding salted fish^a^								
Q1	124,568	401	1.0 (Ref)	0.23	1.0 (Ref)	0.28	1.0 (Ref)	0.24
Q2	125,269	363	0.91 (0.79–1.05)		0.94 (0.80–1.12)		0.91 (0.76–1.09)	
Q3	123,993	321	0.78 (0.67–0.90)		0.79 (0.66–0.94)		0.76 (0.63–0.92)	
Q4	122,517	408	0.96 (0.84–1.11)		0.96 (0.81–1.14)		0.98 (0.82–1.17)	
Q5	118,491	374	0.87 (0.75–1.00)		0.89 (0.74–1.06)		0.84 (0.69–1.02)	
Per 50 g/day increase			0.97 (0.90–1.04)		0.34 (0.04–2.53)		0.26 (0.03–2.42)	
Salted fish^b^								
Q1	122,185	280	1.0 (Ref)	< 0.001	1.0 (Ref)	0.006	1.0 (Ref)	0.04
Q2	125,008	379	1.45 (1.24–1.69)		1.36 (1.12–1.65)		1.21 (0.99–1.50)	
Q3	124,915	377	1.39 (1.19–1.63)		1.29 (1.06–1.57)		1.22 (0.99–1.50)	
Q4	123,374	410	1.49 (1.28–1.74)		1.43 (1.18–1.73)		1.35 (1.10–1.65)	
Q5	120,595	422	1.49 (1.28–1.73)		1.43 (1.18–1.75)		1.30 (1.05–1.61)	
Per 50 g/day increase			1.07 (1.00–1.14)		1.19 (1.03–1.38)		1.17 (0.99–1.38)	
n-3 PUFAs-rich fish^c^								
Q1	124,886	370	1.0 (Ref)	0.34	1.0 (Ref)	0.42	1.0 (Ref)	0.17
Q2	126,196	371	0.94 (0.82–1.09)		0.94 (0.79–1.12)		0.95 (0.79–1.14)	
Q3	124,501	365	0.90 (0.78–1.04)		0.91 (0.76–1.09)		0.90 (0.74–1.09)	
Q4	122,110	376	0.92 (0.79–1.06)		0.87 (0.73–1.05)		0.85 (0.69–1.03)	
Q5	118,383	386	0.92 (0.79–1.06)		0.95 (0.83–1.08)		0.89 (0.77–1.03)	
Per 50 g/day increase			0.98 (0.90–1.07)		0.95 (0.84–1.08)		0.90 (0.78–1.03)	
Marine n-3 PUFAs								
Q1	123,745	310	1.0 (Ref)	0.51	1.0 (Ref)	0.32	1.0 (Ref)	0.22
Q2	124,197	354	1.02 (0.87–1.19)		1.08 (0.90–1.30)		1.15 (0.95–1.40)	
Q3	123,867	402	1.05 (0.90–1.22)		1.08 (0.90–1.31)		1.10 (0.90–1.34)	
Q4	123,709	392	0.97(0.83–1.13)		0.96 (0.80–1.16)		0.99 (0.80–1.21)	
Q5	119,936	408	0.98 (0.84–1.14)		0.97 (0.80–1.18)		0.97 (0.79–1.20)	
Per 1 g/day increase			0.99 (0.93–1.07)		0.97 (0.89–1.07)		0.95 (0.86–1.06)	

**Model 1**: adjusted for age and public health centre area; **Model 2**: adjusted for metabolic equivalent tasks (MET), alcohol consumption, smoking status, body mass index (BMI), history of diabetes mellitus, history of gastric ulcer, hypertension medication, family history of gastric cancer, total energy, meat consumption, vegetable consumption, fruit consumption, and salted food, in addition to the variables adjusted in Model 1

**Sensitivity analysis**: adjusted for variables in Model 2, excluding gastric cancer cases within 3 years of follow-up

*Q* quintile, *HR* hazards ratio, *CI* confidence intervals, *n* number of cases

^a^Adjusted for salted fish consumption in addition to the variables in Model 2

^b^Adjusted for unsalted fish consumption in addition to the variables in Model 2

^c^Adjusted for lean fish in addition to the variables in Model 2

**Fish and shellfish**: canned tuna, salmon/trout, bonito/tuna, cod/flatfish, sea bream, horse mackerel/sardines, mackerel pike/mackerel, chikuwa (Japanese fish cake), kamaboko (Japanese cured surimi (minced fish paste)), salted fish, salted fish roe, dried fish, eel, squid, octopus, prawn, short-necked clam, and viviparide; **fish**: canned tuna, salmon/trout, bonito/tuna, cod/flatfish, sea bream, horse mackerel/sardines, mackerel pike/mackerel, shirasuboshi, salted fish, dried fish, and eel; **salted fish**: salted pike/mackerel, salted cod/flatfish, salted salmon/trout, salted fish roe, dried fish, and shirasuboshi (dried young sardines), salted fish roe, dried fish, and shirasuboshi; **n-3 PUFAs-rich fish**: salmon/trout, horse mackerel/sardines, mackerel pike/mackerel, eel, and sea bream; **marine n-3 PUFAs**: sum of eicosapentaenoic acid (EPA), docosapentaenoic acid (DPA), and docosahexaenoic acid (DHA) consumption

**Table 3:** Hazard ratios (HRs) and 95% confidence intervals (CIs) of gastric cancer risk by fish and shellfish consumption for women

Quintile (Q) of intake	Person-years	Cases (*n*)	Model 1 HR (95% CI)	*p*-trend	Model 2 HR (95% CI)	*p*-trend	Sensitivity analysis HR (95%CI)	*p*-trend
Fish and shellfish								
Q1	147,477	156	1.0 (Ref)	0.06	1.0 (Ref)	0.53	1.0 (Ref)	0.47
Q2	148,929	175	1.01 (0.82–1.26)		1.04 (0.81–1.33)		1.10 (0.84–1.44)	
Q3	150,079	175	0.94 (0.75–1.17)		0.95 (0.74–1.23)		1.00 (0.76–1.32)	
Q4	149,370	153	0.78 (0.62–0.98)		0.80 (0.62–1.05)		0.84 (0.63–1.12)	
Q5	148,024	174	0.87 (0.70–1.08)		0.98 (0.75–1.29)		0.99 (0.74–1.33)	
Per 50 g/day increase			0.99 (0.94–1.06)		1.03 (0.95–1.12)		1.02 (0.94–1.12)	
Fish								
Q1	147,489	157	1.0 (Ref)	0.03	1.0 (Ref)	0.37	1.0 (Ref)	0.31
Q2	149,416	179	1.03 (0.83–1.27)		0.95 (0.74–1.22)		1.02 (0.78–1.33)	
Q3	149,425	171	0.92 (0.74–1.14)		0.93 (0.73–1.20)		1.00 (0.76–1.31)	
Q4	149,743	156	0.79 (0.63–0.99)		0.80 (0.61–1.04)		0.82 (0.62–1.09)	
Q5	147,817	170	0.83 (0.37–1.04)		0.91 (0.69–1.19)		0.92 (0.69–1.23)	
Per 50 g/day increase			0.98 (0.92–1.05)		1.00 (0.92–1.09)		0.98 (0.90–1.08)	
Fish excluding salted fish^a^								
Q1	147,557	161	1.0 (Ref)	0.03	1.0 (Ref)	0.57	1.0 (Ref)	0.68
Q1	149,541	186	1.15 (0.93–1.41)		1.14 (0.89–1.47)		1.24 (0.95–1.62)	
Q2	149,325	163	0.99 (0.80–1.23)		1.06 (0.82–1.36)		1.13 (0.86–1.49)	
Q3	149,118	175	1.04 (0.84–1.28)		1.08 (0.84–1.40)		1.16 (0.88–1.52)	
Q4	146,689	146	0.83 (0.67–1.04)		0.94 (0.72–1.24)		0.98 (0.73–1.31)	
Per 50 g/day increase			0.94 (0.85–1.03)		0.43 (0.02–7.93)		0.52 (0.02—11.7)	
Salted fish^b^								
Q1	146,363	10	1.0 (Ref)	0.11	1.0 (Ref)	0.21	1.0 (Ref)	0.31
Q2	149,438	180	1.70 (1.34–2.16)		1.27 (0.96–1.69)		1.30 (0.96–1.77)	
Q3	150,041	189	1.75 (1.38–2.21)		1.40 (1.06–1.84)		1.51 (1.13–2.03)	
Q4	149,736	176	1.59 (1.25–2.02)		1.25 (0.95–1.66)		1.30 (0.96–1.76)	
Q5	148,301	178	1.53 (1.20–1.94)		1.33 (1.00–1.77)		1.33 (0.96–1.76)	
Per 50 g/day increase			1.08 (1.02–1.13)		1.15 (1.01–1.32)		1.16 (1.00–1.33)	
n-3 PUFAs-rich fish^c^								
Q1	149,707	161	1.0 (Ref)	0.11	1.0 (Ref)	0.43	1.0 (Ref)	0.36
Q1	149,615	159	0.91 (0.73–1.13)		0.87 (0.67–1.13)		0.90 (0.68–1.19)	
Q2	149,612	191	1.06 (0.86–1.31)		1.07 (0.84–1.37)		1.13 (0.87–1.48)	
Q3	149,226	159	0.84 (0.68–1.05)		0.87 (0.67–1.13)		0.89 (0.67–1.18)	
Q4	146,719	163	0.86 (0.69–1.07)		0.89 (0.67–1.17)		0.89 (0.66–1.19)	
Per 50 g/day increase			0.98 (0.88–1.08)		0.99 (0.86–1.14)		0.92 (0.78–1.08)	
Marine n-3 PUFAs								
Q1	146,725	145	1.0 (Ref)	0.03	1.0 (Ref)	0.07	1.0 (Ref)	0.07
Q1	148,183	162	0.93 (0.74–1.16)		0.93 (0.71–1.21)		1.00 (0.75–1.32)	
Q2	149,091	180	0.94 (0.75–1.18)		0.95 (0.73–1.24)		0.99 (0.75–1.32)	
Q3	150,408	181	0.88 (0.70–1.10)		0.89 (0.68–1.16)		0.88 (0.66–1.18)	
Q4	148,682	165	0.78 (0.62–0.99)		0.79 (0.60–1.04)		0.81 (0.60–1.09)	
Per 1 g/day increase			0.94 (0.85–1.05)		0.94 (0.83–1.06)		0.91 (0.80–1.04)	

**Model 1**: adjusted for age and public health centre area; **Model 2**: adjusted for metabolic equivalent tasks (MET), alcohol consumption, smoking status, body-mass index (BMI), history of diabetes mellitus, history of gastric ulcer, hypertension medication, family history of gastric cancer, total energy, meat consumption, vegetable consumption, fruit consumption, and salted food, in addition to variables adjusted in Model 1

**Sensitivity analysis**: adjusted for variables in Model 2, excluding gastric cancer cases within 3 years of follow-up

*Q* quintile, *HR* hazards ratio, *CI* confidence intervals, *n* number of cases

^a^Adjusted for salted fish consumption in addition to variables in Model 2

^b^Adjusted for unsalted fish consumption in addition to variables in Model 2

^c^Adjusted for lean fish in addition to variables in Model 2

**Fish and shellfish**: canned tuna, salmon/trout, bonito/tuna, cod/flatfish, sea bream, horse mackerel/sardines, mackerel pike/mackerel, chikuwa (Japanese fish cake), kamaboko (Japanese cured surimi (minced fish paste)), salted fish, salted fish roe, dried fish, eel, squid, octopus, prawn, short-necked clam, and viviparide; **fish**: canned tuna, salmon/trout, bonito/tuna, cod/flatfish, sea bream, horse mackerel/sardines, mackerel pike/mackerel, shirasuboshi, salted fish, dried fish, and eel; **salted fish**: salted pike/mackerel, salted cod/flatfish, salted salmon/trout, salted fish roe, dried fish, and shirasuboshi (dried young sardines), salted fish roe, dried fish, and shirasuboshi; **n-3 PUFAs-rich fish**: salmon/trout, horse mackerel/sardines, mackerel pike/mackerel, eel, and sea bream; **marine n-3 PUFAs**: sum of eicosapentaenoic acid (EPA), docosapentaenoic acid (DPA), and docosahexaenoic acid (DHA) consumption

We further analysed the association between consumption of fish and shellfish, fish, fish excluding salted fish, salted fish, n-3 PUFAs-rich fish, and marine n-3 PUFAs based on anatomical region of the tumour. For proximal gastric cancer (*n* = 218 men and 68 women), none of the fish and shellfish categories, for both men and women, were associated with cancer risk, except for women in the highest quintile of salted fish consumption (HR for fifth quintile versus first quintile 3.02 (95% CI 1.03–8.81); *p*-trend < 0.10) (data not shown). For distal gastric cancer (*n* = 1219 men and 552 women), consumption of salted fish increased the cancer risk for men, as in the findings from the main analysis (HR for fifth quintile versus first quintile 1.68 (95% CI 1.31–2.14); *p*-trend < 0.001) (data not shown). No clear associations were observed for other fish and shellfish or with marine n-3 PUFAs with distal gastric cancer risk.

### Association between fish and shellfish consumption and gastric cancer risk considering H. pylori infection and atrophic gastritis status

17,583 subjects (6192 men and 11,391 women) had information on *H. pylori* infection status and atrophic gastritis, among whom 482 cases of gastric cancer (288 men and 194 women) were identified during follow-up. The baseline characteristics of subjects in the subgroup analysis were similar to those in the main analysis. Tables 4 and 5 show gastric cancer risk from consumption of fish and shellfish, fish, fish excluding salted fish, salted fish, n-3 PUFAs-rich fish, and marine n-3 PUFAs for men and women, taking *H. pylori* infection and atrophic gastritis into consideration. There were only 1,511 women who drank alcohol; therefore, we modified the alcohol consumption categories to never/former and current for women’s analysis. We observed similar findings to those of the main analysis; an increased gastric cancer risk trend with salted fish consumption for men in the analysis not taking *H. pylori* or atrophic gastritis into consideration (HR for fifth quintile versus first quintile 1.62 (95% CI 0.98–2.65); *p-*trend 0.04) (Table 4). For the analysis by *H. pylori* and atrophic gastritis status, we could not observe an association for *H. pylori − /* AG − due to lack of gastric cancer cases (n = 23 men and eight women). For men, the association between salted fish and gastric cancer risk diminished once *H. pylori* infection and atrophic gastritis status were taken into consideration. For women, similar to the main analysis, none of the fish and shellfish categories considered in our study were associated with gastric cancer risk.

**Table 4:** Hazard ratios (HRs) and 95% confidence intervals (CIs) of gastric cancer risk by quintile of fish and shellfish consumption by *H. pylori* infection or atrophic gastritis (AG) for men

	Overall				*H. pylori* +				AG +				*H. pylori* + and/or AG +			
Quintile (Q) of intake	Person- years	Cases (n)	HR (95% CI)	*p*-trend	Person- years	Cases (n)	HR (95% CI)	*p*-trend	Person- years	Cases (n)	HR (95% CI)	*p*-trend	Person- years	Cases (n)	HR (95% CI)	*p*-trend
Fish and shellfish																
Q1	16,583	54	1.0 (Ref)	0.34	10,950	48	1.0 (Ref)	0.40	5966	36	1.0 (Ref)	0.23	11,410	49	1.0 (Ref)	0.29
Q2	16,336	57	1.25 (0.80–1.98)		11,335	52	1.28 (0.79–2.07)		6492	42	1.31 (0.66–1.95)		11,944	56	1.10 (0.62–1.92)	
Q3	16,381	57	1.02 (0.63–1.64)		11,526	53	1.03 (0.63–1.70)		6447	45	1.10 (0.63–1.90)		11,914	54	1.10 (0.63–1.93)	
Q4	16,162	70	1.39 (0.89–2.19)		11,604	65	1.48 (0.93–2.38)		6439	52	1.49 (0.88–2.54)		12,139	69	1.52 (0.89–2.62)	
Q5	16,020	50	0.80 (0.48–1.34)		11,757	47	0.82 (0.48–1.40)		7379	35	0.68 (0.37–1.25)		12,303	49	0.69 (0.37–1.29)	
Fish																
Q1	16,660	57	1.0 (Ref)	0.32	11,090	50	1.0 (Ref)	0.35	5852	38	1.0 (Ref)	0.21	11,532	51	1.0 (Ref)	0.18
Q2	16,458	49	0.83 (0.53–1.32)		11,446	45	0.86 (0.53–1.40)		6494	39	0.84 (0.49–1.43)		12,208	49	0.80 (0.46–1.39)	
Q3	16,123	63	0.90 (0.71–1.41)		11,304	60	0.97 (0.61–1.54)		6677	47	0.81 (0.47–1.37)		11,847	60	0.85 (0.50–1.45)	
Q4	16,208	68	1.09 (0.71–1.69)		11,649	62	1.16 (0.74–1.84)		6516	49	1.11 (0.67–1.86)		12,139	66	1.11 (0.65–1.87)	
Q5	16,034	51	0.72 (0.44–1.16)		11,682	48	0.73 (0.44–1.21)		7183	37	0.63 (0.36–1.11)		12,184	51	0.60 (0.33–1.08)	
Fish excluding salted fish^a^																
Q1	16,782	45	1.0 (Ref)	0.07	10,441	40	1.0 (Ref)	0.11	5075	28	1.0 (Ref)	0.20	11,130	41	1.0 (Ref)	0.18
Q2	16,518	43	0.74 (0.48–1.13)		10,988	40	0.75 (0.48–1.17)		6078	36	0.88 (0.53–1.44)		11,409	42	0.81 (0.48–1.35)	
Q3	16,085	67	0.63 (0.40–0.99)		11,608	61	0.69 (0.43–1.10)		6764	51	0.72 (0.42–1.24)		12,162	63	0.77 (0.44–1.32)	
Q4	16,094	64	0.78 (0.50–1.20)		11,972	59	0.81 (0.52–1.28)		7029	45	0.82 (0.48–1.41)		12,408	63	0.79 (0.46–1.37)	
Q5	16,003	69	0.58 (0.36–0.94)		12,163	65	0.59 (0.35–1.17)		7776	50	0.68 (0.38–1.20)		12,601	68	0.63 (0.34–1.15)	
Salted fish^**^																
Q1	16,433	68	1.0 (Ref)	0.04	11,287	61	1.0 (Ref)	0.09	3835	45	1.0 (Ref)	0.58	11,781	62	1.0 (Ref)	0.57
Q2	16,554	59	0.95 (0.57–1.58)		12,031	54	0.97 (0.57–1.68)		6715	48	1.05 (0.57–1.93)		12,503	59	1.00 (0.53–1.88)	
Q3	16,170	53	1.49 (0.93–2.40)		11,091	49	1.39 (0.84–2.29)		6487	40	1.44 (0.81–2.56)		11,636	50	1.41 (0.78–2.57)	
Q4	16,204	57	1.35 (0.82–2.21)		11,451	54	1.25 (0.75–2.11)		6526	39	1.04 (0.56–1.94)		11,849	55	0.97 (0.51–1.86)	
Q5	16,076	51	1.62 (0.98–2.65)		11,266	47	1.51 (0.90–2.53)		6577	38	1.28 (0.69–2.35)		11,895	51	1.27 (0.89–2.06)	
n-3 PUFAs-rich fish^c^																
Q1	16,754	50	1.0 (Ref)	0.12	10,733	45	1.0 (Ref)	0.10	5846	36	1.0 (Ref)	0.79	11,308	46	1.0 (Ref)	0.61
Q2	16,559	69	1.33 (0.86–2.07)		11,832	64	1.29 (0.81–2.04)		6209	44	1.15 (0.68–1.96)		12,297	66	1.11 (0.65–1.92)	
Q3	16,315	57	0.88 (0.54–1.43)		11,653	52	0.87 (0.52–1.46)		6823	46	0.87 (0.49–1.55)		12,097	56	0.85 (0.47–1.52)	
Q4	15,986	62	1.13 (0.70–1.83)		11,540	58	1.14 (0.69–1.87)		7043	42	1.08 (0.61–1.89)		12,040	59	1.05 (0.59–1.89)	
Q5	15,867	50	0.75 (0.43–1.30)		11,413	46	0.70 (0.39–1.25)		6801	42	0.95 (0.50–1.79)		11,969	50	0.86 (0.44–1.67)	
Marine n-3 PUFAs																
Q1	16,537	55	1.0 (Ref)	0.06	11,503	51	1.0 (Ref)	0.10	6158	33	1.0 (Ref)	0.49	11,897	52	1.0 (Ref)	0.55
Q1	16,211	63	1.14 (0.73–1.78)		10,879	55	1.09 (0.74–1.61)		6276	47	1.33 (0.77–2.29)		11,436	60	1.26 (0.71–2.21)	
Q2	16,275	59	1.05 (0.67–1.68)		11,713	57	1.00 (0.67–1.49)		6680	45	1.15 (0.66–2.03)		12,222	58	1.21 (0.68–2.15)	
Q3	16,366	62	0.87 (0.53–1.42)		11,378	57	0.98 (0.65–1.48)		6603	44	0.93 (0.50–1.71)		11,949	59	0.95 (0.51–1.77)	
Q4	15,952	48	0.67(0.39–1.16)		11,609	44	0.71 (0.45–1.13)		6944	40	0.96 (0.51–1.80)		12,087	47	0.95 (0.50–1.82)	

Model adjusted for age, public health centre area, metabolic equivalent tasks (METs), alcohol consumption, smoking status, body-mass index (BMI), history of diabetes mellitus, history of gastric ulcer, hypertension medication, family history of gastric cancer, total energy, meat consumption, vegetable consumption, fruit consumption, and salted food

*Q* quintile, *HR* hazards ratio, *CI* confidence intervals; n: number of cases, *H. pylori* +  *H. pylori positive*, *AG* +  atrophic gastritis, *H. pylori* + and/or *AG* +  *H. pylori positive* and/or atrophic gastritis

^a^Adjusted for salted fish consumption in addition to variables in the model

^b^Adjusted for unsalted fish consumption in addition to variables in the model

^c^Adjusted for lean fish in addition to variables in the model

**Fish and shellfish**: canned tuna, salmon/trout, bonito/tuna, cod/flatfish, sea bream, horse mackerel/sardines, mackerel pike/mackerel, chikuwa (Japanese fish cake), kamaboko (Japanese cured surimi (minced fish paste)), salted fish, salted fish roe, dried fish, eel, squid, octopus, prawn, short-necked clam, and viviparide; **fish**: canned tuna, salmon/trout, bonito/tuna, cod/flatfish, sea bream, horse mackerel/sardines, mackerel pike/mackerel, shirasuboshi, salted fish, dried fish, and eel; **salted fish**: salted pike/mackerel, salted cod/flatfish, salted salmon/trout, salted fish roe, dried fish, and shirasuboshi (dried young sardines), salted fish roe, dried fish, and shirasuboshi; **n-3 PUFAs-rich fish**: salmon/trout, horse mackerel/sardines, mackerel pike/mackerel, eel, and sea bream; **marine n-3 PUFAs**: sum of eicosapentaenoic acid (EPA), docosapentaenoic acid (DPA), and docosahexaenoic acid (DHA) consumption

**Table 5:** Hazard ratios (HRs) and 95% confidence intervals (CIs) of gastric cancer risk by quintiles of fish and shellfish consumption by *H. pylori* infection or atrophic gastritis (AG) for women

	Overall				*H. pylori* +				AG +				*H. pylori* + and/or AG +			
Quintile (Q) of intake	Person- years	Cases (*n*)	HR (95%CI)	*p*-trend	Person- years	Cases (*n*)	HR (95%CI)	*p*-trend	Person- years	Cases (n)	HR (95%CI)	*p*-trend	Person- years	Cases (n)	HR (95%CI)	*p*-trend
Fish and shellfish																
Q1	32,383	41	1.0 (Ref)	0.19	21,052	35	1.0 (Ref)	0.15	13,336	28	1.0 (Ref)	0.47	21,954	38	1.0 (Ref)	0.19
Q2	32,307	42	1.17 (0.70–1.96)		21,776	40	1.25 (0.73–2.14)		13,044	27	0.96 (0.52–1.76)		22,569	41	1.19 (0.71–2.00)	
Q3	32,803	42	0.86 (0.50–1.50)		22,375	42	0.96 (0.54–1.69)		13,726	23	0.60 (0.31–1.19)		23,207	42	0.89 (0.51–1.55)	
Q4	32,495	44	1.19 (0.71–2.01)		22,101	38	1.12 (0.64–1.97)		13,112	35	1.33 (0.74–2.40)		22,918	41	1.15 (0.67–1.97)	
Q5	32,361	25	0.66 (0.35–1.24)		21,520	22	0.66 (0.33–1.29)		13,794	17	0.64 (0.96–1.02)		22,266	24	0.66 (0.35–1.27)	
Fish																
Q1	32,536	34	1.0 (Ref)	0.13	21,430	30	1.0 (Ref)	0.02	13,716	23	1.0 (Ref)	0.43	22,355	32	1.0 (Ref)	0.11
Q2	32,294	53	1.53 (0.91–2.59)		21,442	49	1.44 (0.91–2.29)		12,813	33	1.40 (0.75–2.62)		22,170	51	1.67 (0.98–2.86)	
Q3	32,433	40	1.13 (0.65–1.99)		22,221	38	1.03 (0.63–1.68)		13,878	27	1.15 (0.60–2.18)		23,147	40	1.20 (0.68–2.11)	
Q4	32,705	41	0.20 (068–2.10)		22,171	38	1.01 (0.61–1.65)		13,100	30	1.37 (0.72–2.62)		22,988	40	1.22 (0.69–2.17)	
Q5	32,381	26	0.75 (0.39–1.44)		21,560	22	0.62 (0.34–1.10)		13,506	17	0.79 (0.36–1.71)		22,256	23	0.75 (0.38–1.48)	
Fish excluding salted fish^a^																
Q1	32,719	23	1.0 (Ref)	0.85	20,783	19	1.0 (Ref)	0.78	12,139	14	1.0 (Ref)	0.83	21,873	22	1.0 (Ref)	0.85
Q2	32,299	40	1.57 (0.95–2.60)		21,571	39	1.89 (1.10–3.24)		13,540	29	1.54 (0.84–2.80)		22,490	39	1.72 (1.02–2.90)	
Q3	32,329	56	1.17 (0.67–2.05)		21,778	53	1.21 (0.66–2.24)		13,999	39	1.06 (0.53–2.10)		22,524	55	1.28 (0.72–2.29)	
Q4	32,574	41	1.39 (0.80–2.42)		21,973	38	1.69 (0.93–3.04)		13,146	29	1.50 (0.78–2.88)		22,703	41	1.58 (0.89–2.80)	
Q5	32,428	34	0.97 (0.95–2.60)		22,718	28	1.18 (0.59–2.35)		14,189	19	1.09 (0.50–2.35)		23,326	29	1.09 (0.55–2.13)	
Salted fish^b^																
Q1	32,719	23	1.0 (Ref)	0.47	21,438	33	1.0 (Ref)	0.09	13,553	25	1.0 (Ref)	0.25	22,286	36	1.0 (Ref)	0.13
Q2	32,299	40	1.11 (0.60–2.05)		22,018	49	1.14 (0.60–2.16)		14,204	36	1.19 (0.57–2.51)		22,819	49	1.01 (0.55–1.88)	
Q3	32,329	56	1.84 (1.04–3.27)		21,843	34	1.71 (0.94–3.14)		12,936	23	1.75 (0.86–3.58)		22,713	38	1.64 (0.92–2.92)	
Q4	32,574	41	1.03 (0.55–1.94)		22,131	38	0.93 (0.48–1.81)		13,018	28	1.07 (0.49–2.31)		22,879	39	0.93 (0.50–1.75)	
Q5	32,428	34	1.00 (0.51–1.93)		21,173	22	0.73 (0.35–1.49)		13,194	17	0.83 (0.36–1.91)		21,997	23	0.73 (0.37–1.46)	
n-3 PUFAs-rich fish^c^																
Q1	32,539	37	1.0 (Ref)	0.83	21,356	31	1.0 (Ref)	0.92	13,114	21	1.0 (Ref)	0.37	22,290	34	1.0 (Ref)	0.90
Q2	32,730	37	0.87 (0.51–1.49)		21,610	37	1.04 (0.59–1.83)		13,442	26	1.03 (0.53–1.98)		22,331	37	0.97 (0.56–1.68)	
Q3	32,246	51	1.20 (0.71–2.03)		21,786	48	1.33 (0.76–2.32)		13,340	34	1.48 (0.77–2.85)		22,726	50	1.29 (0.75–2.22)	
Q4	32,507	39	1.21 (0.70–2.09)		22,710	34	1.21 (0.67–2.18)		13,824	26	1.44 (0.73–2.83)		23,468	37	1.24 (0.70–2.18)	
Q5	32,326	30	0.86 (0.46–1.64)		21,361	27	0.99 (0.50–1.95)		13,293	23	1.36 (0.63–2.96)		22,100	28	0.93 (0.48–1.82)	
Marine n-3 PUFAs																
Q1	32,163	38	1.0 (Ref)	0.24	21,725	33	1.0 (Ref)	0.30	14,090	26	1.0 (Ref)	0.86	22,643	36	1.0 (Ref)	0.26
Q2	32,291	44	1.26 (0.74–2.14)		22,185	43	1.29 (0.74–2.23)		13,158	27	1.12 (0.59–2.13)		22,950	44	1.24 (0.72–2.11)	
Q3	32,360	42	1.15 (0.67–1.99)		21,334	38	1.15 (0.65–2.06)		13,251	27	1.21 (0.64–2.29)		22,308	40	1.11 (0.64–1.94)	
Q4	32,586	38	1.02 (0.58–1.80)		21,851	35	1.02 (0.56–1.87)		13,232	25	0.93 (0.47–1.85)		22,467	36	0.98 (0.55–1.75)	
Q5	32,689	32	0.78 (041–1.47)		21,587	28	0.81 (0.41–1.58)		13,219	25	1.03 (0.50–2.10)		22,404	30	0.79 (0.41–1.50)	

Model adjusted for age, public health centre area, metabolic equivalent tasks (MET), alcohol consumption, smoking status, body-mass index (BMI), history of diabetes mellitus, history of gastric ulcer, hypertension medication, family history of gastric cancer, total energy, meat consumption, vegetable consumption, fruit consumption, and salted food

*Q* quintile, *HR* hazards ratio, *CI* confidence intervals, *n* number of cases, *H. pylori* + :*H. pylori positive*, *AG* +  atrophic gastritism *H. pylori* + and/or *AG* +  *H. pylori positive* and/or atrophic gastritis

^a^Adjusted for salted fish consumption in addition to variables in the model

^b^Adjusted for unsalted fish consumption in addition to variables in the model

^c^Adjusted for lean fish in addition to variables in the model

**Fish and shellfish**: canned tuna, salmon/trout, bonito/tuna, cod/flatfish, sea bream, horse mackerel/sardines, mackerel pike/mackerel, chikuwa (Japanese fish cake), kamaboko (Japanese cured surimi (minced fish paste)) salted fish, salted fish roe, dried fish, eel, squid, octopus, prawn, short-necked clam, and viviparide; **fish**: canned tuna, salmon/trout, bonito/tuna, cod/flatfish, sea bream, horse mackerel/sardines, mackerel pike/mackerel, shirasuboshi, salted fish, dried fish, and eel; **salted fish**: salted pike/mackerel, salted cod/flatfish, salted salmon/trout, salted fish roe, dried fish, and shirasuboshi (dried young sardines), salted fish roe, dried fish, and shirasuboshi; **n-3 PUFAs-rich fish**: salmon/trout, horse mackerel/sardines, mackerel pike/mackerel, eel, and sea bream; **marine n-3 PUFAs**: sum of eicosapentaenoic acid (EPA), docosapentaenoic acid (DPA), and docosahexaenoic acid (DHA) consumption

## Discussion

In this study, we aimed to determine the association between fish and shellfish consumption and gastric cancer risk among a Japanese population. We found an increase in gastric cancer risk with salted fish consumption for both men and women. When *H. pylori* infection and atrophic gastritis status were considered, none of the fish and shellfish consumption categories, for both men and women, were associated with gastric cancer risk.

While limited studies have assessed the association between gastric cancer risk and fish or n-3 PUFAs, our study findings are in line with previous studies [11, 12]. In a Japanese setting, the Japan Collaborative Cohort Study have evaluated associations between 33 food items and gastric cancer risk. While the study found no clear association between fish and gastric cancer, a non-significant increase in risk was observed among women in the third-highest fish consumption category (HR 1.62 (95% CI 0.95 – 2.75)) [28].

Previous JPHC study have also shown an increase in gastric cancer risk with salted fish consumption for men [8]. The current study, with a longer follow-up period and larger sample size, observed an increased  gastric cancer risk for both men and women. High salt concentration in the intra-gastric region could destroy the mucosal barrier, causing inflammation and damage. This would in turn lead to symptoms such as diffuse erosion and degeneration of mucosa that could induce proliferous changes and enhance food-derived carcinogenetic effects [29]. Mucosa damage could also enhance *H. pylori* colonisation though gastric mucosa damage, increasing the risk of gastric cancer [30, 31].

In the main analysis, we also observed a borderline significant decreasing trend in gastric cancer risk for marine n-3 PUFAs for women. One possible mechanism of how marine n-3 PUFAs could reduce gastric cancer risk is through suppression of inflammation. Dietary n-3 PUFAs can be metabolised into prostaglandins, thromboxanes, hydroxyeicosatetraenoic acids and leukotrienes, which possess anti-inflammatory and immune-regulatory characteristics through enzymatic activity [32]. n-3 PUFAs can also be metabolised into resolvins and protectins, which also have anti-inflammatory and immune-regulatory characteristics [33, 34].

The significant associations in our main analysis disappeared in the subgroup analysis which considered *H. pylori* infection and atrophic gastritis status. This suggests that *H. pylori* infection is the strongest risk factor for gastric cancer onset.

The major strength of this study is its use of a prospective cohort of a large sample of subjects recruited from the general population. Given the long follow-up period (average 15 years), high follow-up questionnaire response rate, low loss to follow-up, and provision of cancer cases from population-based cancer registries, we believe that gastric cancer cases were sufficiently identified. We used gastric cancer incidence as an end point instead of death to directly measure gastric cancer risk. We conducted a sensitivity analysis which excluded gastric cancer cases diagnosed within 3 years of follow-up, which further validates our findings. Having information on atrophic gastritis and *H. pylori* infection using a blood test allowed us to adjust for the strongest gastric cancer risk factor.

There were several limitations in our study. Most subjects of the JPHC studies were recruited in non-metropolitan areas, which may have led to a geographically biased result that may not be applicable to the general population. Atrophic gastritis and *H. pylori* status were only available for a subgroup of subjects, and due to the limited number of cases we were unable to look at the association for those who tested negative to both *H. pylori* and atrophic gastritis. However, given that *H. pylori* prevalence among those who were born before 1950 was over 80% in Japan [35], we believe our findings are valid. FFQ validity for fish and marine n-3 PUFAs intake was moderate, which may have introduced measurement error and non-deferential misclassification, biasing the HRs towards the null. We did not have information on how the fish was cooked, and thus were not able to perform the analysis based on the cooking method. Lastly, while we were not able to obtain the date of cancer diagnosis for those who the diagnosis was notified through death certificate notification [DCN, *n* = 159 (5.9%)] or death certification only [DCO, *n* = 114 (4.2%)], given the low percentage of DCN and DCO cases, we believe we were able to identify sufficient number of cancer cases that represent the population.

Despite the limitations, we believe we have provided new insights into the association between fish and shellfish consumption and gastric cancer risk. There may be other mechanisms through which fish consumption may be protective of gastric cancer. More studies are needed to understand the true association between fish, shellfish, and gastric cancer risk, especially in Asian countries, where seafood consumption is high.

## Data Availability

For information on data availability, please refer to this link: https://epi.ncc.go.jp/en/jphc/805/8155.html.

## Abbreviations

95% CI: 95% Confidence intervals
AG: Atrophic gastritis
DHA: Docosahexaenoic acid
DPA: Docosapentaenoic acid
EPA: Eicosapentaenoic acid
HR: Hazard ratio
*H. pylori*: *Helicobacter pylori*
The JPHC Study: The Japan Public Health Center-based Prospective Study
PHC: Public health center
PUFAs: Polyunsaturated fatty acids
